# DNA Oxidation and Excision Repair Pathways

**DOI:** 10.3390/ijms20236092

**Published:** 2019-12-03

**Authors:** Tae-Hee Lee, Tae-Hong Kang

**Affiliations:** Department of Biological Science, Dong-A University, Busan 49315, Korea; thlee@donga.ac.kr

**Keywords:** reactive oxygen species (ROS), nucleotide excision repair (NER), base excision repair (BER), oxidative DNA damage

## Abstract

The physiological impact of the aberrant oxidation products on genomic DNA were demonstrated by embryonic lethality or the cancer susceptibility and/or neurological symptoms of animal impaired in the base excision repair (BER); the major pathway to maintain genomic integrity against non-bulky DNA oxidation. However, growing evidence suggests that other DNA repair pathways or factors that are not primarily associated with the classical BER pathway are also actively involved in the mitigation of oxidative assaults on the genomic DNA, according to the corresponding types of DNA oxidation. Among others, factors dedicated to lesion recognition in the nucleotide excision repair (NER) pathway have been shown to play eminent roles in the process of lesion recognition and stimulation of the enzyme activity of some sets of BER factors. Besides, substantial bulky DNA oxidation can be preferentially removed by a canonical NER mechanism; therefore, loss of function in the NER pathway shares common features arising from BER defects, including cancer predisposition and neurological disorders, although NER defects generally are nonlethal. Here we discuss recent achievements for delineating newly arising roles of NER lesion recognition factors to facilitate the BER process, and cooperative works of BER and NER pathways in response to the genotoxic oxidative stress.

## 1. Introduction

The integrity of the genome is endlessly threatened by reactive oxygen species (ROS) formed in living cells as metabolic byproducts. Therefore, maintenance of genomic integrity against ROS is a prerequisite for proper cell function and, hence, maintaining homeostasis. Essential cellular functions, such as oxidative phosphorylation and lipid peroxidation, produce ROS that can induce oxidative DNA damage. It has been estimated that as many as ten thousand oxidation reactions harm DNA per each human cell per day [1], which eventually produces a plethora of non-bulky (purine/pyrimidine base oxidations) and bulky (crosslinks, strand breaks, and cyclic bases) DNA lesions. These lesions, if not timely repaired, can interfere with essential DNA metabolisms, including transcription, recombination, and replication, which ultimately can give rise to unfavorable outcomes like cellular senescence and mutagenesis.

Unlike other cellular macromolecules, damaged DNA cannot be replaced and solely depends on repair to remain intact. In order to counteract oxidative DNA lesions, base excision repair (BER) is thought to be the primary pathway to remove non-bulky modifications formed largely on the bases of the DNA, including 8-oxo-7,8-dihydroguanine (8-oxoG), the most prevailing purine base oxidation with highly mutagenic potential, which is sometimes compared with thymine glycol, the most frequent pyrimidine one with relatively limited mutation frequency [2]. Meanwhile, nucleotide excision repair (NER), the most versatile DNA repair system in human cells, has been demonstrated to actively operate to neutralize DNA oxidation, especially with bulky oxidative lesions such as cyclopurines [3]. When damage is extensive, these repair processes are accompanied by cell-cycle checkpoint activation, which provides cells with sufficient time to either complete the repair or initiate apoptosis [4,5,6,7].

## 2. DNA Excision Repairs and Implication on Human Health

**Figure 1:**
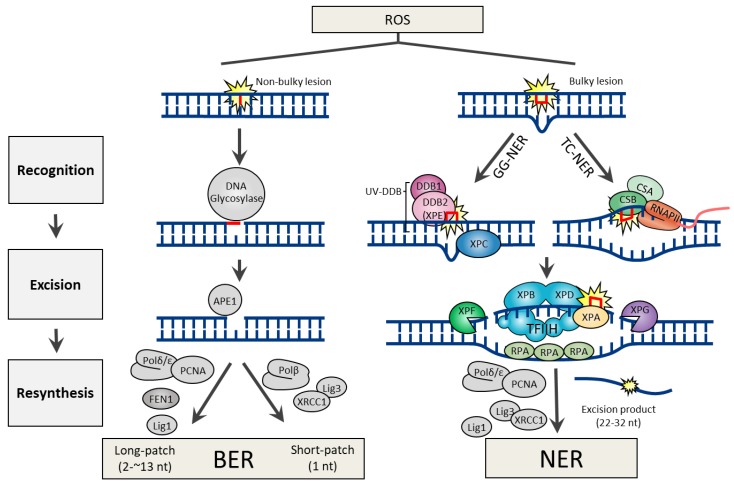
DNA excision repair mechanisms for bulky or non-bulky DNA lesions caused by reactive oxygen species (ROS).

DNA lesions arising from the ROS attack can generate both non-bulky (non-helix distorting) and bulky (helix distorting) lesions. In human cells, BER and NER are the two DNA excision repair pathways responsible for the removal of non-bulky and bulky DNA lesions, respectively. Both repair pathways share three common steps, which include 1) lesion recognition, 2) excision of damaged nucleotide, and 3) resynthesis using error-free DNA polymerases (Figure 1).

### 2.1. Base Excision Repair

Many of the genes involved in BER are highly conserved from bacteria to humans [8], indicating that BER is a fundamental repair pathway in most living organisms. The BER pathway is specialized to fix non-bulky single-base lesions in the form of small chemical modifications, including oxidation, alkylation, and deamination damage. Base modifications are pro-mutagenic and/or cytotoxic, depending on how they interfere with the template function of the DNA during replication and transcription. To initiate the procedure, BER employs a specific DNA glycosylase for lesion recognition and elimination of the damaged base. Although every DNA glycosylase has a distinct structure and substrate specificity, all glycosylases share a common mode of action for damage recognition; 1) flipping the affected base out of the DNA helix, which facilitates a sensitive detection of even minor base modifications, 2) catalyzing the cleavage of an N-glycosidic bond, releasing a free base and creating an abasic site (apurinic/apyrimidinic site or AP site). DNA glycosylases can be either monofunctional or bifunctional. Monofunctional DNA glycosylases possess only the glycosylase activity, which includes UNG (uracil-N glycosylase), SMUG1 (single-strand-specific monofunctional uracil DNA glycosylase), MBD4 (methyl-binding domain glycosylase 4), TDG (thymine DNA glycosylase), MYH (MutY homolog DNA glycosylase), MPG (methylpurine glycosylase). In contrast, bifunctional DNA glycosylases have an intrinsic 3′ AP lyase activity accompanying with the glycosylase activity, which includes OGG1 (8-oxoguanine DNA glycosylase), NTH1 (endonuclease ΙΙΙ-like), and NEIL1 (endonuclease VΙΙΙ-like glycosylase). The processes following DNA glycosylase are common to the BER mechanism irrespective of the identity of the glycosylase. Base removal by a DNA glycosylase generates an AP site in DNA, which is then further processed by AP endonuclease 1 (APE1), which cleaves the DNA backbone 5′ to the abasic site, generating a 3′-hydroxyl and a 5′-2-deoxyribose-5′-phosphate (5′-dRP). DNA polymerase β (Polβ) utilizes the 3′-hydroxyl to fill the gap through template-directed synthesis. Depending on the number of nucleotides added, either short-patch (where a single nucleotide is replaced) or long patch BER (where 2-13 nucleotides are synthesized) pathways operate to complete the repair process. In short-patch BER, the intrinsic dRP-lyase activity of Polβ removes the 5′-dRP. The addition of more than one nucleotide (up to 13) constitutes long-patch BER and requires the assistance of flap endonuclease 1 (FEN1) to remove the displaced 5′-flap structure (Figure 1). 

### 2.2. General Features of BER Defect

Genetic loss or mutation in key genes of the BER process, such as *APE1* [9], *Polβ* [10], *FEN1* [11], or the *DNA ligase 3* [12], has been shown to have embryonic lethality in mice, while the phenotype of DNA glycosylase disruptions in mice is usually rather moderate, the only known exception being the TDG, which was essential for embryonic development in mice [13,14].

Neurons encounter particularly high levels of oxidative stress because of the high metabolic rate required to support their electrical and synaptic functions. Thus, the integrity and capacity of systems that repair oxidative DNA damage would be expected to be critical for the survival and proper function of neurons, particularly under conditions of increased oxidative stress that occur during catastrophic pathological conditions, including ischemic stroke. In a study with a mouse model of focal cerebral ischemic brain injury performed on normal and *OGG1*^−/−^ mice, the potential role of OGG1 in ameliorating the detrimental effect of oxidative DNA damage to neurons was evaluated. The results indicate that after cerebral ischemia, the accumulation of brain oxidative DNA base lesions was significantly greater in *OGG1*-deficient mice, and was associated with greater brain damage and poorer behavioral outcomes, revealing an important role for OGG1 in brain BER capacity, which contributes to neuronal survival after experimental stroke [15].

Neurons from *OGG1*-deficient mice are sensitive to oxidative stress and reduced OGG1 levels have also been associated with Alzheimer’s disease (AD) [16]. Indeed, levels of expression of UNG, OGG1, and Polβ are lower in brain tissue from patients with AD than in brain tissue from age-matched controls without AD [17]. *Polβ*^+/−^ heterozygote displayed impaired synaptic and cognitive functions, linking the loss of heterozygous BER function in the progression of AD [18].

While a mouse lacking *UNG* develops B-cell lymphomas, there is often not a clear phenotype in a mouse for a single DNA glycosylase mutation, presumably due to the substrate redundancy. For instance, the *OGG1*-deficient mice are viable and fertile without any visible phenotype [19,20], which suggests overlapping activities for the repair of 8-oxoG lesions. However, interestingly, additional deletion of *MYH* in *OGG1*-deficient mice predisposes 65.7% of mice to tumors, predominantly lung and ovarian tumors, and lymphomas [21]. 8-oxoG indeed is a common substrate of MYH and OGG1. 

### 2.3. Nucleotide Excision Repair

NER is famous for the unique repair pathway in humans to remove photolesions produced by UV radiation (sun exposure) that mainly forms cyclobutane pyrimidine dimer (CPD), a non-bulky lesion and pyrimidine-(6,4)-pyrimidone product (6-4PP), a bulky lesion. Besides, it also efficiently eliminates an extremely broad range of structurally unrelated DNA lesions, including bulky chemical adducts and intrastrand crosslinks [22]. The basis of the versatility of NER originates that it circumvents recognition of the lesion itself, instead, the lesion recognizing NER factors detects the presence of unpaired single-stranded DNA opposite the damaged strand [23]. 

Owing to the distinct damage recognition events, NER mechanisms can be further specified into two subpathways, global genome NER (GG-NER) and transcription-coupled NER (TC-NER). The former is responsible for eliminating lesions throughout the whole genome, while the latter is for those in the transcribing strand of active genes. During GG-NER, XPC (xeroderma pigmentosum C) or UV-DDB (UV-damaged DNA binding protein; a heterodimeric complex with DDB1 and DDB2) initiate the recognition of the damage. Structural analysis showed certain lesions, such as CPD, which do not significantly distort the DNA helix, are first recognized by DDB2 (also known as XPE) to extrude the lesions into its binding pocket, and thereby create a kink that is now recognized by XPC [24]. If the lesion is in a transcribed gene, it is sensed as a blockage to RNA polymerase II (RNAPII) and requires Cockayne syndrome B (CSB) and CSA to initiate the TC-NER process [25]. Regardless of the damage recognition mechanisms, the downstream events are conserved in both NER mechanisms. Damage verification is executed by XPA and helix unwinding is carried out by TFIIH (complexed with the XPB and XPD helicases). Lesion excision is catalyzed by the structure-specific endonucleases XPF and XPG, which incise the damaged strand at 5′ and 3′ from the lesion, respectively, which promotes releasing out of the lesion containing 22–32 nt-long oligomers. Final DNA gap-filling synthesis and ligation are executed by the replication proteins proliferating cell nuclear antigen (PCNA), Polδ, Polε, and DNA ligase 1 or XRCC1 (X-ray repair cross-complementing protein 1)–DNA ligase 3 complex (Figure 1). 

### 2.4. General Features of NER Defect

Hereditary mutations in NER-associated genes are nonlethal and associated with disorders that are characterized by UV sensitivity and cancer predisposition, such as XP, CS, and trichothiodystrophy (TTD) [26]. XP comprises seven complementation groups (*XPA*-*XPG*) with defective GG-NER. Five of these groups also exhibit defective TC-NER, whereas *XP-C* and *XP-E* patients are TC-NER-proficient. The XP patient shows hypersensitivity on minimal sun exposure, pigmentary accumulations at exposed skin regions, and multiple early age skin cancers. Progressive neuronal degeneration is also observed in approximately one-third of XP cases, generally after the appearance of cutaneous signs [27]. However, unlike GG-NER-deficient XP patients, CS patients belong to one of two complementation groups (*CS-A* or *CS-B*), and those that are completely defective in TC-NER are not cancer-prone but exhibit a drastic reduction in life span [28]. In addition, CS patients display a number of neurological and developmental abnormalities as well as hypersensitivity to sun exposure. Although the molecular basis that leads to the diverse features of CS remains largely unknown, a reduced ability of cells to relieve oxidative stress has been proposed to be a leading cause [29,30,31]. Since cells from CS patients were found to be hypersensitive to oxidative DNA damage, a role for the CS proteins in the response to oxidized bases has been proposed [32]. Mutations in the *CSB* account for the majority of CS cases [33]. 

Clinical heterogeneity in disorders with NER mutations opens the question of whether defects in this pathway are solely due to impaired repair of helix-distorting DNA lesions. XP patients along with TCR defects (caused by some specific alterations in XPB, XPD, and XPG) present, besides increased skin cancer risk, accelerated neurodegeneration, and CS symptoms (XP/CS). The causative relationship between mutations and the CS clinical features in XP/CS cases is complex and must not only involve the NER defects but also the other functions of the NER proteins. Several studies have demonstrated that transcription impairment, oxidative repair, and energy metabolism alteration, as well as genotoxic stress, may explain the combined XP/CS phenotype [34,35]. Neuronal death might be due to accumulated endogenous damage, and indeed a growing body of evidence indicates that NER proteins participate in the processing of oxidative DNA lesions that are produced by the normal cell metabolism. The role of NER proteins in different pathways might explain the heterogeneity in disorders with NER mutations [36].

## 3. Facilitated BER Kinetics by the NER Lesion Recognition Factors

The role of NER factors in the control of oxidative DNA damage is reviewed elsewhere [36]. Intriguingly, the key lesion recognition factors CSB (for TC-NER) and UV-DDB and XPC (for GG-NER) are implicated in facilitating the enzyme activity of key BER factors, such as DNA glycosylases and APE1, thereby they contribute to the protection of cells against non-bulky oxidative DNA lesion, such as 8-oxoG. Surprisingly; however, this initial recruitment of NER factors does not trigger the downstream NER process, which includes the lesion verification by the XPA and TFIIH complex [37]. 

### 3.1. XPC

XPC, the main lesion sensor in GG-NER presents as a heterotrimeric complex with HR23B (human RAD23 homolog B) and centrin 2 proteins. HR23B stabilizes the XPC protein [38], and the XPC-HR23B heterodimer is sufficient to reconstitute the cell-free NER reaction [39], whereas centrin 2 appears to facilitate the damage-specific DNA binding activity of the XPC complex [40]. This complex binds to various types of bulky lesions, thus triggering GG-NER and it also participates in the repair of non-bulky base lesions. Consequently, *XPC* deficiency not only results in decreased GG-NER but has also been linked to disturbed redox homeostasis due to the accumulating DNA oxidations. The XPC complex functionally interacts with OGG1, MPG, and TDG that initiate BER of oxidation, alkylation, and deamination products, respectively. D’Errico et al. show that the XPC–HR23B complex acts as a cofactor in the BER pathway via mediating the OGG1 loading and turnover kinetics, thereby freeing OGG1 to react with remaining lesions [41]. While analyzing the biochemical properties behind mutations found in *XP-C* patients, a critical single amino acid substitution at position 334 (P334H) weakening the interaction with OGG1 is defined. Cells from this patient exhibit low efficiency of UV-induced unscheduled DNA synthesis and a decreased OGG1 cleavage activity, indicating that the OGG1 activity is stimulated by XPC through direct interaction with its N-terminal part that encompasses the P334 surrounding region. This patient is also one of the rare XP-C patients who exhibit neurological symptoms [42].

The interaction of the MPG protein with XPC-HR23B proteins stimulates the DNA glycosylase activity and is correlated to the increased binding affinity of the MPG-HR23B protein complex for the substrate [43]. Biochemical studies demonstrate that XPC-HR23B also participates in BER of guanine/thymine or guanine/uridine mismatches, which are mainly derived from hydrolytic deamination of 5-methylcytosines or cytosines, respectively. The BER of these mismatches is initiated by TDG. The XPC complex is capable of stimulating TDG activity by promoting the release of TDG following the excision of the mismatched T base [44]. In addition, XPC stimulates the glycosylase activities of TDG and SMUG1, both of which interact physically with XPC [45].

### 3.2. UV-DDB

DNA in eukaryotes is packaged in tandemly arrayed nucleosomes that, together with numerous DNA- and nucleosome-associated enzymes and regulatory factors, make up chromatin [46]. Because DNA lesions that result from ROS can occur both within and outside of nucleosomes, the repair efficiency is necessarily dependent on the accessibility and structural requirements for enzyme catalysis to overcome the hindrance presented by the location of a DNA lesion. Therefore, it is reasonable that chromatin remodeling in the vicinity of damaged DNA is critical for enabling efficient repair and the subsequent repackaging of DNA into nucleosomes. Indeed several studies have shown that DNA lesions in the nucleosome are limiting access for glycosylases and APE1. 

While XPC is required for GG-NER, it has little or no affinity for CPD lesions and does not recognize 6-4PP in the context of chromatin [47]. XPC recruitment to chromatin is facilitated by the UV-DDB (DDB1 and DDB2) complex [48]. In the absence of DDB2, XPC remains localized to 6-4PP and to a lesser extent to CPDs with substantially delayed kinetics [47]. UV-DDB, as part of CUL4A-RBX E3 ubiquitin ligase, has been shown to modify core histones around the sites of UV lesions [49]. 

The glycosylases remain bound to their AP site product until displaced by APE1, a step which is rate-limiting for most mammalian glycosylases [50]. Recently, Jang et al. defined the specific roles of UV-DDB in the early steps of BER mechanisms and proposed that UV-DDB is a general sensor of DNA damage in both NER and BER pathways, facilitating damage recognition in the context of chromatin. Specifically, they find that UV-DDB facilitates both OGG1 and APE1 strand cleavage and promotes Polβ-mediated gap-filling activity by 30-fold. The single-molecule real-time imaging technique reveals the dynamic interaction between UV-DDB and OGG1 or APE1, which facilitates turnover rates of OGG1 and APE1 from DNA, hence increasing BER capacity. Furthermore, in light of a novel chemoptogenetic approach, the dynamic recruitment of UV-DDB to locally-induced 8-oxoG sites in telomeric regions of DNA is detected in vivo [51]. 

### 3.3. CSB

A transcription elongation factor CSB (also known as ERCC6), in a complex with RNAPII, strongly binds to RNAPII when it is blocked by a bulky lesion and alters nucleosome structure near its occupancy sites by wrapping the DNA around the protein itself to trigger TC-NER [52]. However, 8-oxoG lesions, which only cause minor helix-distortions, do not block RNAPII elongation unless processed by its specific glycosylase OGG1, implying that transcription-coupled BER, if it exists, may not be directly triggered by stalled RNAPII on the oxidative lesions themselves [53].

Evidence for the role of CSB in the BER process has been provided by several groups, which report that cellular extracts from CSB null cells demonstrate reduced incision activity of oxidative DNA lesions in vitro [54,55,56,57]. CSB (but not downstream core NER factors) accumulates at sites of locally-induced oxidative damage in vivo, in a transcription-dependent manner, with similar kinetics as the OGG1 [37]. An interesting finding pinpoints that lysine (K) 991 in CSB is subject to being ubiquitinated and this ubiquitination selectively occurs in response to oxidative damage, shedding new light on the critical role of CSB on the discrimination of the different repair choices [58].

PARP1 is believed to stimulate the BER process by recruiting the DNA repair apparatus to the single-strand breaks and is found in complex with the BER protein XRCC1, DNA ligase 3, and Polβ. Thorslund et al. demonstrate that CSB is a novel substrate for PARP1 poly-ADP-ribosylation and that this modification inhibits the catalytic ATPase activity of CSB, while the meaning of poly-ADP-ribosylation of CSB remains to be answered [59]. In the meantime, it was reported that poly-ADP-ribosylated PARP1 is required for retention of CSB at sites of oxidative DNA damage, so that CSB promotes PARP1 displacement from damaged DNA to facilitate BER [60,61].

## 4. Oxidative DNA Damages that Can Be Readily Repaired by a Canonical NER

### 4.1. Bulky Lesions

ROS-induced covalent modifications to DNA encompass tandem base modification (a form of intrastrand crosslinks), purine 5′,8-cyclonucleosides, interstrand cross-links, and DNA–protein crosslinks [62,63]. Biochemical studies demonstrated that these lesions could markedly block DNA replication and transcription and that these lesions are repaired by the NER pathway [63,64]. A tandem base lesion G [8–5m]T is structurally similar to the CPD [65]. The XPA-deficient human brain and mouse liver contain higher levels of G[8–5m]T but not ROS-induced simple base lesions [66]. 5′,8-cyclo-2′-deoxyadenosine (cdA) and 5′,8-cyclo-2′-deoxyguanosine (cdG) are tandem lesions produced by the attack of hydroxyl radicals to the purine bases of DNA [67]. These lesions are also repaired primarily by the NER pathway [68].

Covalent DNA–protein crosslinks (DPCs, also known as protein adducts) represent an important class of DNA damage that may be produced according to the different mechanisms by certain enzymes that form covalent reaction intermediates with DNA, chemotherapeutics, and various endogenous and exogenous sources [69]. DPCs are highly toxic as they interfere with nearly all chromatin-based processes. Model studies have shown that 2-deoxyribonolactone (dL), an oxidized abasic site, is able to undergo crosslink formation with enzymes of the BER pathway, including Polβ [70]. Bifunctional DNA glycosylases possessing AP lyase activity, such as OGG1 and NTH1, have also been shown to form DPC in vitro with both dL and with its β–elimination product, butenolide [54]. It has been postulated that proteolytic digestion of the covalently-bound enzyme to DNA could be implicated in the initial repair process prior to being completed by NER enzymes [71].

The abasic site aldehyde is reactive and may progress to an interstrand crosslink (ICL) to a purine on the opposing strand [72,73]. Since the ICL lesion affects both strands of the DNA it is considered as a highly toxic DNA lesion that prevents transcription and replication by inhibiting DNA strand separation. So far, NER, translesion DNA synthesis, homologous recombination, and the Fanconi anemia pathway are identified to be involved in ICL repair in a coordinated fashion [74].

### 4.2. Non-Bulky Lesions

The 8-oxoG lesions tend to be easily further oxidized to form stereoisomeric spiroiminodihydantoin (Sp) and guanidinohydantoin (Gh) lesions [75,76]. These are still small DNA lesions that are generally efficiently recognized and excised by DNA glycosylases. In vitro analysis demonstrates the hydantoin lesions are not only excellent BER substrates but are also excised by the NER complex prepared as cell-free extract [77]. Recently, Shafirovich et al., using a cell-based assay, demonstrated that the BER and NER pathways compete with one another in intact human cells and can catalyze both Gh and Sp lesions [78]. The relative contribution of either process in intact cells depends on the local availability of the primary NER and BER factors that recognize and bind to the same lesions in a competitive fashion.

In addition, abundant 8-oxoG and thymine glycol lesion, both are canonical BER substrates, can be removed by NER in vitro as fast as CPD lesion [79]. To investigate 8-oxoG repair in intact living cells, a laser-assisted procedure to locally inflict oxidative DNA lesions was developed by Menoni et al. [37]. In light of this in vivo study, strong and very rapid recruitment of CSB and XPC to 8-oxoG lesions was observed. Interestingly, CSB exhibited a direct transcription-dependent repair of oxidative lesions associated with different RNA polymerases (RNAPI and RNAPII), but not involving other NER proteins.

The repair of AP lesions takes place predominantly by the APE1-mediated BER pathway. However, among chemically heterogeneous AP lesions formed in DNA, some are resistant to APE1 and thus refractory to BER [80]. Using reporter constructs accommodating stable APE1-resistant AP lesions, Kitsera et al. demonstrates that NER efficiently removes BER-resistant AP lesions and significantly enhances the repair of APE1-sensitive ones as well [81].

## 5. Concluding Remarks

The impact of ROS-induced oxidative DNA damage on human health and counteracting DNA excision repair pathways is discussed. Inevitable DNA oxidation reactions can interrupt essential DNA metabolisms and, thereafter, can evoke various stressful cellular responses including replication- and transcription stress response. Therefore, timely relief of the stresses by error-free DNA excision repair systems (BER and NER) is essential to maintain genomic integrity and proper cell function. While the causal relationship between human disorder and the loss of function of a specific NER gene is relatively clear, fewer connections have been made between impaired BER and human diseases. This is likely due to the multitude of backup systems like NER in the removal of small non-bulky lesions.

In light of the state-of-the-art technologies, including CRISPR/CAS9, optogenetics, and next-generation sequencing, great progress has been made towards understanding the precise mechanisms underlying the enhanced BER activity by NER-initiating factors. Besides, through the studies aimed at elucidating the complex mechanisms that underlie the NER- or BER-related phenotypes, now we are getting a better understanding of the fundamentals of diseases such as cancer, aging, and neuropathology. One of the major future challenges is to translate these valuable findings into human health benefits in the clinic for individualized cancer therapies based on precision medicine, and for the development of a well-aging strategy as well. Synthetic lethal strategies targeting DNA repair defects, using small molecule inhibitors such as PARP inhibitors, are shedding light on cancer treatment with low adverse-effects and more tumor-selective killing [82]. Small molecule modulators of NER/BER activity or specific protein–protein interactions could be new tools for chemical biology studies and might lead to new therapeutic approaches [83].

## Abbreviations

5′-dRP	5′-2-deoxyribose-5′-phosphate
6-4PP	Pyrimidine-(6,4)-pyrimidone product
8-oxoG	8-oxo-7,8-dihydroguanine
Abasic site	Apurinic/apyrimidinic site or AP site
AD	Alzheimer’s disease
APE1	AP endonuclease 1
BER	Base excision repair
cdA	5′,8-cyclo-2′-deoxyadenosine
cdG	5′,8-cyclo-2′-deoxyguanosine
CPD	Cyclobutane pyrimidine dimer
CSB	Cockayne syndrome B
dL	2-deoxyribonolactone
DPCs	DNA–protein crosslinks
FEN1	Flap endonuclease 1
GG-NER	Global genome NER
Gh	Guanidinohydantoin
HR23B	Human RAD23 homolog B
ICL	Interstrand crosslink
MBD4	Methyl-binding domain glycosylase 4
MPG	Methylpurine glycosylase
MYH	MutY homolog DNA glycosylase
NEIL1	Endonuclease VIII-like glycosylase
NER	Nucleotide excision repair
NTH1	Endonuclease III-like glycosylase
OGG1	8-oxoguanine DNA glycosylase
PCNA	Proliferating cell nuclear antigen
Polβ	DNA polymerase β
RNAPII	RNA polymerase II
ROS	Reactive oxygen species
SMUG1	Single-strand-specific monofunctional uracil DNA glycosylase
Sp	Spiroiminodihydantoin
TC-NER	Transcription-coupled NER
TDG	Thymine DNA glycosylase
TFIIH	Transcription Factor II H; complexed with the XPB and XPD helicases
TTD	Trichothiodystrophy
UNG	Uracil-N glycosylase
UV-DDB	UV-damaged DNA binding protein; a heterodimeric complex with DDB1 and DDB2
XPC	Xeroderma pigmentosum C
XRCC1	X-ray repair cross-complementing protein 1
